# Bitten by a Centipede

**Published:** 1886-02

**Authors:** 


### BITTEN BY A CENTIPEDE.

A curious and very remarkable, and at the same time most distressing case of insect poisoning occurred in this city on Saturday last. In the afternoon of that day Miss Irene Tay, a well known society young lady, residing at No. 1005 Leavenworth street near Pine, was visiting a friend on the same street west of Devisadero. It was the season of housecleaning at the friend's house. The carpets were up, and while Miss Tay was walking through one of the rooms she felt something creep up one of her legs and bite her. The bite was followed by an insufferable burning sensation, and she decided to return home immediately. On leaving the car of the California-street line the young lady was obliged to walk about a half block to reach her home, and this she did with great difficulty, as her leg was paining her frightfully, and was partially paralyzed. On reaching her home and removing her clothing, by the assistance of her mother and sister, an enormous centipede fell to the floor. Members of the family applied arnica to the injured limb, but it afforded little relief. The member was much inflamed and swollen. A druggist was sent for, but he afforded little relief, and finally Dr. Eckle, the family physician, arrived. The doctor pronounced the case a very serious one. The young lady was suffering so severely that it was feared she would die, but under the care of the physician she recovered, so that now she is thought to be out of danger. The centipede was three and a half inches long and supplied with innuuerable legs and tenticles. An attempt was made to kill the venemous reptile by pouring ammonia on it and scorching it with a flat iron, but it seemed to suffer little inconvenience from these attentions. It was finally placed in a bottle and shown to the physician, who at once announced its dangerous character.—*San Fancisco Call.*

